# Reference values of 25-hydroxyvitamin D revisited: a position statement from the Brazilian Society of Endocrinology and Metabolism (SBEM) and the Brazilian Society of Clinical Pathology/Laboratory Medicine (SBPC)

**DOI:** 10.20945/2359-3997000000305

**Published:** 2020-10-08

DOI: 10.20945/2359-3997000000258

Arch Endocrinol Metab. 2020;64(4):462-78

Where you read (TITLE):

Reference values of 25-hydroxyvitamin D revisited: a position statement from the Brazilian Society of Endocrinology and Metabolism (SBEM) and the Brazilian Society of Clinical Pathology/Laboratory Medicine (SBPC)

Should read:

Reference values of 25-hydroxyvitamin D revisited: a position statement from the Brazilian Society of Endocrinology and Metabolism (SBEM) and the Brazilian Society of Clinical Pathology/Laboratory Medicine ( **SBPC/ML** )

Where you read (TEXT):

These assays include a first step in which 25(OH)D is dissociated from its carrier proteins. In a second step, the 25(OH)D in the sample competes with an analogue for the same sites of the ligand's assay **[anti-25(OH)D or DBP antibodies]** .

Should read:

These assays include a first step in which 25(OH)D is dissociated from its carrier proteins. In a second step, the 25(OH)D in the sample competes with an analogue for the same sites of the ligand's assay **[anti-25(OH)D antibodies and DBP]** .

Where you read:

**Table 2:** Clinical conditions and groups that benefit from 25-hydroxyvitamin (25[OH]D) concentrations above 30 ng/mL

Groups	Clinical Conditions
Elderly (> 65 years)	Osteoporosis (primary or secondary)
Pregnant women	Fractures due to fragility
	Metabolic bone diseases (osteomalacia, osteogenesis imperfecta, primary hyperparathyroidism) Secondary hyperparathyroidism
	Sarcopenia
	Recurring falls
	Chronic renal disease
	Malabsorption syndrome
	Liver failure
	Anorexia nervosa
	Cancer

Should read:

**Table 2:** Clinical conditions and groups that benefit from 25-hydroxyvitamin (25[OH]D) concentrations above 30 ng/mL

Groups	Clinical Conditions
Elderly (> 65 years)	Osteoporosis (primary or secondary)
Pregnant women	Fractures due to fragility
	Metabolic bone diseases (osteomalacia, osteogenesis imperfecta, primary hyperparathyroidism) Secondary hyperparathyroidism
	Sarcopenia
	Recurring falls
	Chronic renal disease
	Malabsorption syndrome
	Liver failure
	**Diabetes mellitus type 1**
	Cancer

Where you read:

**Correspondence to:**

Carolina Moreira

Rua Leão Júnior, 285

80060-000 – Curitiba, PR Brasil

carolina.aguiar.moreira@gmail.com

Should read:

Carolina Moreira

**Av Agostinho Leão Júnior, 285**

80060-000 – Curitiba, PR Brasil

carolina.aguiar.moreira@gmail.com

